# Hematite (*α*-Fe_2_O_3_) with Oxygen Defects: The Effect of Heating Rate for Photocatalytic Performance

**DOI:** 10.3390/ma17020395

**Published:** 2024-01-12

**Authors:** Masanori Sakamoto, Ryoga Fujita, Masami Nishikawa, Hideyuki Hirazawa, Yuichi Ueno, Manami Yamamoto, Suzu Takaoka

**Affiliations:** 1 Department of Environmental Materials Engineering, National Institute of Technology (KOSEN), Niihama College, 7-1 Yagumo, Niihama 792-8580, Japan; 2 Department of Materials Science and Bioengineering, Nagaoka University of Technology, 1603-1 Kamitomioka, Nagaoka 940-2137, Japan; 3 Center for Integrated Technology Support, Nagaoka University of Technology, 1603-1 Kamitomioka, Nagaoka 940-2137, Japan

**Keywords:** photocatalyst, hematite, Fe_2_O_3_, defect, nanoparticle

## Abstract

Hematite (*α*-Fe_2_O_3_) emerges as an enticing material for visible-light-driven photocatalysis owing to its remarkable stability, low toxicity, and abundance. However, its inherent shortcomings, such as a short hole diffusion length and high recombination rate, hinder its practical application. Recently, oxygen vacancies (Vo) within hematite have been demonstrated to modulate its photocatalytic attributes. The effects of Vo can be broadly categorized into two opposing aspects: (1) acting as electron donors, enhancing carrier conductivity, and improving photocatalytic performance and (2) acting as surface carrier traps, accelerating excited carrier recombination, and deteriorating performance. Critically, the generation rate, distribution, role, and behavior of Vo significantly differ for synthesis methods due to differences in formation mechanisms and oxygen diffusion. This complexity hampers simplified discussions of Vo, necessitating careful investigation and nuanced discussion tailored to the specific method and conditions employed. Among various approaches, hydrothermal synthesis offers a simple and cost-effective route. Here, we demonstrate a hydrothermal synthesis method for Vo introduction to hematite using a carbon source, where variations in the heating rate have not been previously explored in terms of their influence on Vo generation. The analyses revealed that the concentration of Vo was maximized at a heating rate of 16 °C/min, indicative of a high density of surface defects. With regard to photocatalytic performance, elevated heating rates (16 °C/min) fostered the formation of Vo primarily on the hematite surface. The photocatalytic activity was 7.1 times greater than that of the sample prepared at a low heating rate (2 °C/min). These findings highlight the crucial role of surface defects, as opposed to bulk defects, in promoting hematite photocatalysis. Furthermore, the facile control over Vo concentration achievable via manipulating the heating rate underscores the promising potential of this approach for optimizing hematite photocatalysts.

## 1. Introduction

Hematite (*α*-Fe_2_O_3_) stands out as a promising visible-light-driven photocatalyst due to its low cost, abundance, non-toxicity, high stability, and favorable bandgap (Eg = 2.0–2.2 eV). However, its practical application is constrained by its limited hole diffusion length (2–4 nm), short excitation lifetime (10 ps), and restricted light penetration depth (*α*^−1^ = 118 nm at λ = 550 nm) [1,2,3]. In order to make hematite a good candidate for photocatalytic material, many strategies have been explored, including (1) nanostructure engineering to enhance light absorption, diffusion length, and reaction sites and (2) conjunction with other materials like carbon, metal, and semiconductors to improve carrier transport [1,2,3].

Among these approaches, intentional defect engineering has emerged as a propitious technique for enhancing performance [4,5] because some defects endow the materials with many unique physiochemical properties, which has the potential for designing efficient photocatalysts [6,7,8,9,10,11]. As one of the most common defects, the generation and effect of oxygen vacancy (Vo) in hematite have been explored, such as via (1) heat treatment [4,5], (2) plasma treatment [12], ball-milling [13], and aerosol-assisted chemical vapor deposition [14]. Importantly, the generation rate, distribution, role, and behavior of Vo were significantly different for synthesis methods.

Among the various methods, hydrothermal synthesis is one of the simplest and most cost-effective [15,16]. The investigation of the generation mechanism and behavior of Vo in this method is valuable from the practical standpoint of commercialization. In hydrothermal synthesis, FeOOH is first formed, followed by *α*-Fe_2_O_3_. This contrasts with the electron deposition method, which directly produces *α*-Fe_2_O_3_ from Fe without undergoing a precursor such as FeOOH. According to Chen et al. [6], FeOOH exhibits a complex oxygen diffusion mechanism in hydrothermal synthesis. Specifically, oxygen diffuses in two ways: (1) the internal excess oxygen inside the material escapes in the form of water to the surface, and (2) the oxygen in the air diffuses into the interior of material at high temperatures. These results lead to oxygen ratio enrichment at the surface, as confirmed by XPS etching spectra in the synthesis temperature range of 650–720 °C. However, further understanding is required regarding the following: (1) the influence of heating rate for generating Vo in hydrothermal synthesis and (2) the influence of Vo on the decomposition of dyes in photocatalysts. In addition, synthesis conditions at lower temperatures are desirable from the standpoint of energy conservation.

In this background, we focused on a method of introducing Vo using a carbon source via hydrothermal synthesis, changing the heating rate. Specifically, we added acrylic acid to synthesize solution in the hydrothermal synthesis procedure and synthesized hematite while incorporating polyacrylic acid (PAA). Then, PAA was carbonized by heating at 450 °C in the heating rate range of 2–16 °C/min. Then, Vo was introduced by a reduction reaction with oxygen of hematite. In this method, oxygen atoms do not dissociate from hematite independently but react with carbon to form CO or CO_2_ and dissociate. This is different from the existing methods and mechanisms for generating Vo. Therefore, the distribution of Vo and their behavior on photocatalytic activity require further experiments and considerations.

The optimal condition for Vo introduction was determined to be the addition of 4 mL of acrylic acid followed by heating at 450 °C with a rate of 16 °C/min. Notably, the photocatalyst exhibited 7.1 times higher photocatalytic activity compared to that prepared at a low heating rate (2 °C/min). By clarifying this, the discussion on the generation mechanism and behavior of Vo, which has not been fully understood so far, will progress. This simple hydrothermal synthesis results in higher efficiency not only for hematite but also for other semiconductor photocatalysts.

## 2. Materials and Methods

### 2.1. Preparation of Hematite NPs

All reagents employed in this study were of analytical grade and employed without further purification. Hematite with Vo was synthesized primarily following Zeng’s method [16]. Briefly, 2 mmol of FeCl₃·6H₂O (purity >99.0%, NACALAI TESQUE, INC., Kyoto, Japan) and 4 mmol of urea (purity >99.0%, NACALAI TESQUE, INC., Kyoto, Japan) were dissolved in 46 mL of distilled water under constant stirring. Subsequently, 4 mL of acrylic acid (AA) (purity >99.0%, NACALAI TESQUE, INC., Kyoto, Japan) was incorporated into the resulting yellow solution. The solution was then transferred to a 50 mL Teflon-lined stainless-steel autoclave and heated at 140 °C for 12 h. Following cooling to room temperature, the red gel-like product (encompassing poly acrylic acid (PAA)) was collected via centrifugation, thoroughly washed with distilled water, and dried overnight in an oven at 80 °C. Acrylate monomers undergo a radical polymerization reaction to yield PAA. Other substances undergo a series of chemical transformations to synthesize hematite via *β*-FeOOH as an intermediate reactions as follows [17]:

CO(NH_2_)_2_ + H_2_O → 2NH_3_ + CO_2_,
NH_3_ + H_2_O → NH_4_^+^ + OH^−^
FeCl_3_ + 3OH^−^ → Fe(OH)_3_ + 3Cl^−^
Fe(OH)_3_ → *β*-FeOOH + H_2_O
2*β*-FeOOH → *α*-Fe_2_O_3_ + H_2_O

**Scheme 1:**
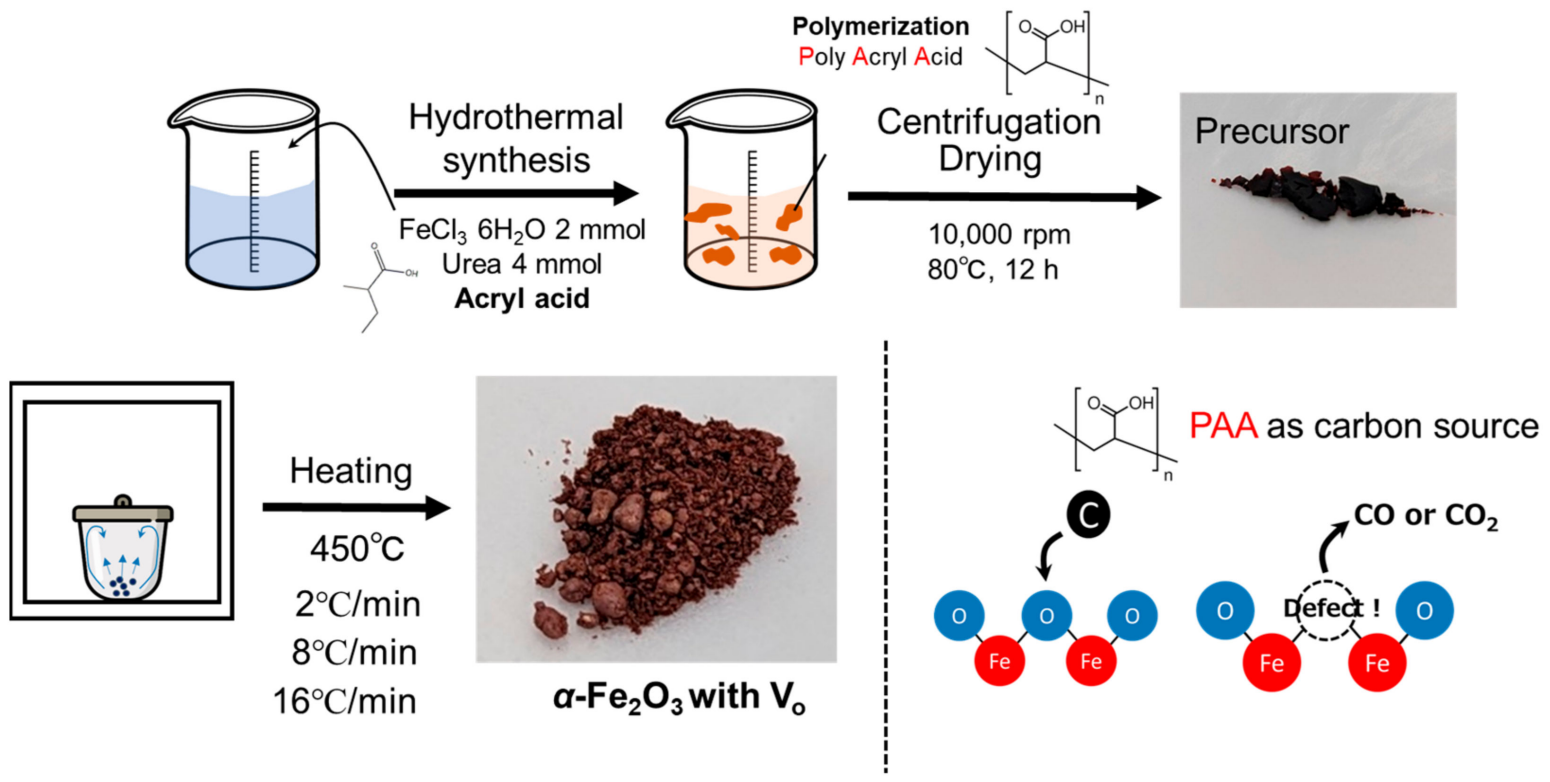
Schematic illustration of the preparation of hematite with Vo.

Finally, the as-prepared precursor was calcined in air at 450 °C for 1.5 h with a heating rate ranging from 2 to 16 °C/min in an electric furnace (FO301, Yamato Scientific Co., Ltd., Tokyo, Japan), which can achieve a maximum heating rate of 16 °C/min as depicted in Scheme 1. This procedure was conducted to induce the carbonization of PAA and to facilitate the removal of oxygen from hematite via the following reaction [18], thereby generating Vo.

Direct reduction: Fe_2_O_3_ + 3C → 2Fe + 3CO
Indirect reduction: Fe_2_O_3_ + 3CO → 2Fe + 3CO_2_
Carbon loss reaction: C + CO_2_ → 2CO

### 2.2. Evaluation of Hematite Nanoparticles

The size, morphology, and atomic composition of hematite nanoparticles (NPs) were analyzed by scanning electron microscopy (SEM) (JSM-7500F, JEOL Ltd., Tokyo, Japan) equipped with an Energy Dispersive X-ray Spectroscopy (EDS) detector. The identification and crystallization of samples were analyzed using X-ray diffraction (XRD) (Rint 2000, Rigaku Corporation, Tokyo, Japan) measurements using Cu K*α* radiation (λ = 1.541 Å), equipped software (PDXL ver. 2.8) to calculate crystallite size and micro-strain, and Fourier Transform Infrared Spectroscopy (FT-IR) (Spectrum TWO, PerkinElmer U.S. LCC., Shelton, CT, USA). X-ray photoelectron spectroscopy (XPS) (Nexsa, Thermo Fisher Scientific Inc., Waltham, MA, USA) characterization was carried out to analyze the surface oxygen defects of samples. The thermogravimetric and differential thermal analysis (TG-DTA) (Thermo plus EVO, Rigaku Corporation, Tokyo, Japan) was performed to investigate the change condition of precursor to hematite samples.

### 2.3. Evaluation of Photocatalytic Performance of Hematite with Vo

The products were used as photocatalysts for the methylene blue (MB) (TCI, Tokyo, Japan) degradation. In all experiments, 10 mg of prepared sample and 0.1 mL of 35% H_2_O_2_ (TCI, Tokyo, Japan) solution were added to 60 mL of MB aqueous solution, which concentration was 1.0 × 10^−5^ M (3.2 mg of MB dissolved in 1L of distilled water). As we will discuss below, this concentration is sufficiently diluted for analysis. After 5 min ultrasonication to prevent the aggregation of NPs in the solution, the dispersive solution was stirred in the dark for 20 min to achieve adsorption equilibrium. The dispersion was then exposed to with visible light irradiation from a white light-emitting diode (SLA-100B, SIGMAKOKI CO.,LTD., Tokyo, Japan). After centrifugation, the MB supernatant was measured by UV-visible spectrophotometer and the change in concentration was evaluated from the absorbance intensity. The irradiation power was 20 mW/cm^2^, measured using a power meter that supports visible light and calculated using the irradiated area of the sensor. The initial concentration of MB before exposure to light was designated as *C*_0_ and the concentration after light irradiation was referred to as *C*. The degradation rate was expressed as the ratio of (*C*/*C*_0_). The reaction rate constant *k* was determined via exponential fitting of these data. According to previous studies, the photodegradation of MB can be regarded as a pseudo-first-order reaction [19], and the reaction kinetics can be expressed as follows if the *C*_0_ is small enough: The following equations were used:

(1) $$\left(\frac{C}{C_{0}}\right)=e^{-kt}\;$$

(2) $$\ln\left(\frac{C_{0}}{C}\right)=kt$$

In addition, the reaction rate constant *k* was recalculated on a per-particle basis (See Supporting Information). The calculation was performed using the average particle size from the histogram and density (*d* = 5.24 g/cm^3^) calculated from the histogram (vide infra).

## 3. Results

**Figure 1:**
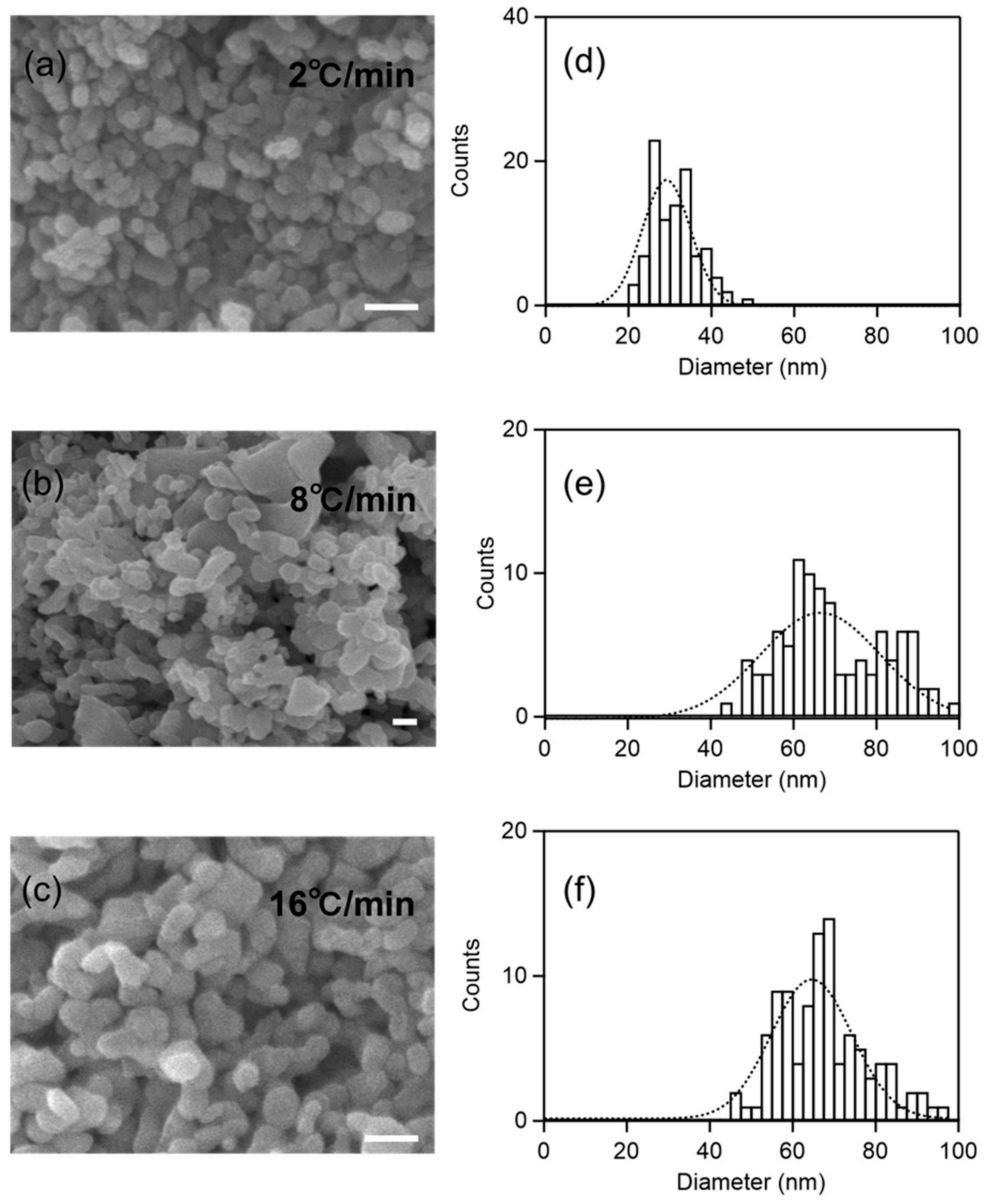
SEM images of hematite NPs (scale bar = 100 nm). The heating rate of (**a**) 2 °C/min, (**b**) 8 °C/min, and (**c**) 16 °C/min. The histogram of each hematite NP. Heating rate of (**d**) 2 °C/min (**e**) 8 °C/min, and (**f**) 16 °C/min respectively. The dotted line indicates the Gauss fitting curve.

Figure 1a–c present representative scanning electron microscopy (SEM) images of the hematite nanoparticles (NPs). These SEM images show that the synthesized hematite NPs are rough, irregular, rock-like shapes. The size distribution of NPs was analyzed using histograms, as shown in Figure 1d–f. The size range of the hematite NPs is *ca.* 30–70 nm. Specifically, the average sizes are 30 ± 5 nm, 70 ± 13 nm, and 67 ± 10 nm, respectively. The size information was later used to calculate the photocatalytic performance per particle.

**Figure 2:**
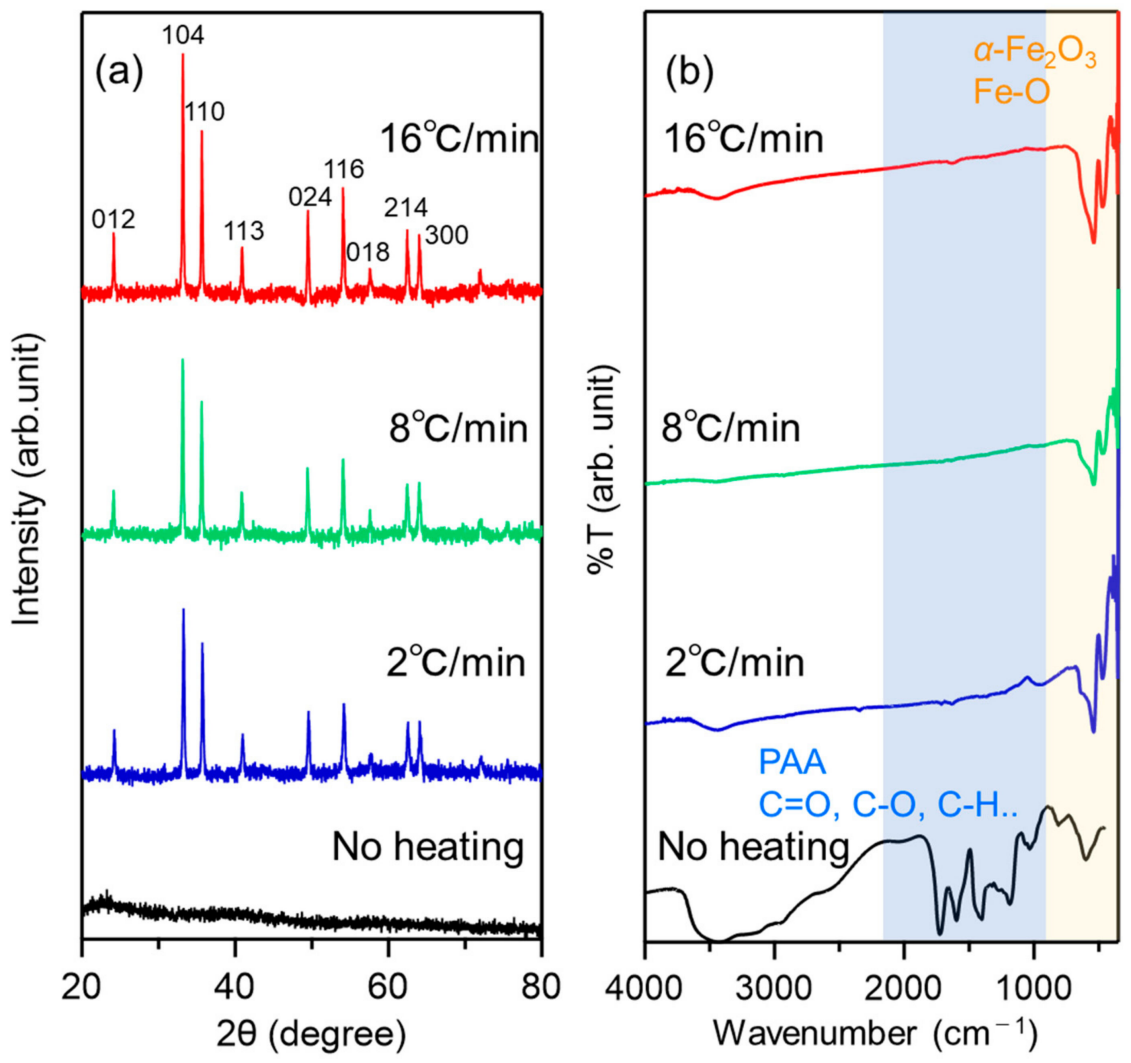
(**a**) XRD and (**b**) FT-IR spectra of prepared samples. The black lines indicate the data of sample before heating at 450 °C. The blue, green, and red lines indicate the data of samples after heating processes at rates of 2°C/min, 8°C/min, and 16°C/min respectively. The blue and yellow areas in (**b**) indicate the peak areas of PAA, and hematite.

Figure 2a shows the XRD patterns of the precursor and the samples heated at 450 °C for 1.5 h. No peaks were observed in the precursor, indicating that hematite was not formed. On the other hand, peaks corresponding to hematite were observed for all heating rates. This suggests that hematite was formed at 450 °C for 1.5 h heating. In addition, no other forms of iron oxide, such as Fe_3_O_4_ or *β*-FeOOH, were detected as impurities. As for Vo, the high concentration of Vo induces a matrix phase change [20,21]. Indeed, previous studies have reported a phase transition from Fe₂O₃ to Fe₃O₄ upon prolonged heat treatment in nitrogen due to continuous oxygen loss [20]. However, as evidenced by the XRD data presented in Figure 2a, the samples prepared at varying heating rates in this work did not exhibit such phase transformation. This suggests that the generated V_O_ concentration remained within the optimal range, preventing significant alteration of the hematite crystal structure. Next, we analyzed the XRD data using the Williamson–Hall plot to calculate the crystallite size and micro-strain. The results show that the crystallite size was 19.4 nm (2 °C/min), 30.5 nm (8 °C/min), and 39.8 nm (16 °C/min), respectively. Therefore, the NPs were found to be polycrystalline, and the tendency of size change was generally consistent with that observed by SEM. The micro-strain was negligible for all prepared samples. Thus, the heating rates used in our study did not induce stress or strain in the crystal due to defects. Furthermore, we ascertained that 450 °C is the optimal temperature for this method via the performance of preliminary experiments at varying temperatures and subsequent XRD pattern analysis (see Supporting Information, Figure S1).

Figure 2b shows the FT-IR spectra of the precursor and the heated samples. In the precursor before heating, the representative peaks corresponding to CO double bond and COO-stretching bonds at 1408 and 1733 cm^−1^, scissor and bending vibrations of -CH_2_, and CHCO groups at 1451 and 1418 cm^−1^ from PAA were observed [22]. On the other hand, these peaks were absent in the heated samples, and instead, peaks corresponding to Fe-O bonds were observed at 470 and 540 cm^−1^ [23]. These FT-IR results suggest that PAA was completely decomposed after heating and that the synthesis of hematite was successful without impurities.

**Figure 3:**
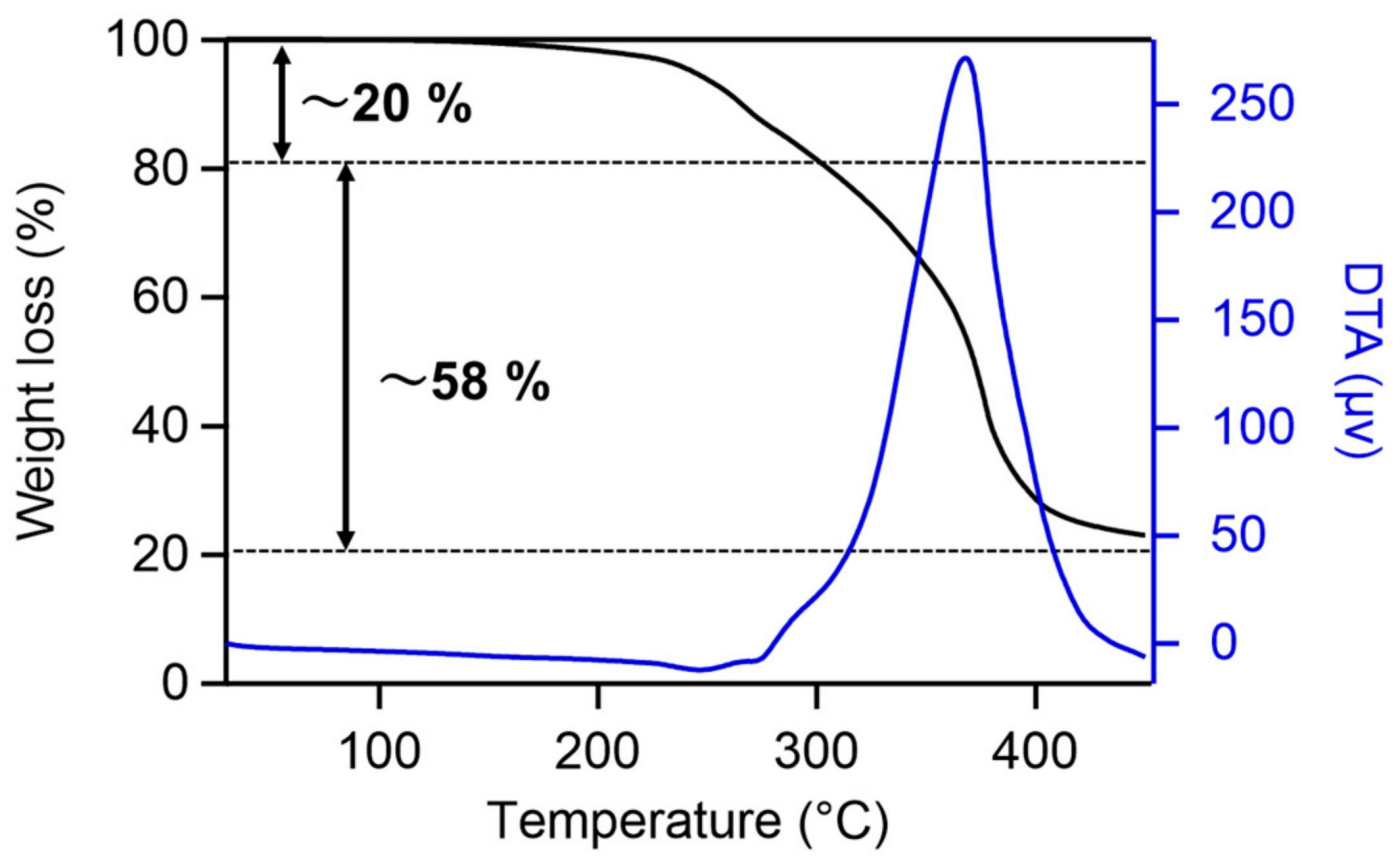
Representative TG-DTA analysis for precursor (AA 4 mL, heating rate: 16 °C/min). The black line represents the thermogravimetric analysis, and blue line represents corresponding differential thermal analysis curve.

Figure 3 shows the results of TG-DTA measurements. Up to 300 °C, a mass loss of 20% was observed. These are thought to be due to the loss of organic impurities containing water. On the other hand, a large exothermic peak was observed between 300 and 400 °C. These are thought to be due to the continuous overlap of exothermic peaks associated with the carbonization of PAA, the reduction reaction of oxygen from hematite, and the combustion and loss of carbon. After these reactions, the mass decreased by 58%. It can be seen that the amount of hematite after the reaction is less than 20% of the precursor. The result was the same tendency as previously reported [16]. As shown in the DTA curve, the reaction was completed at approximately 450 °C. Therefore, 450 °C is the appropriate heating temperature. In fact, when heating at 350 °C, the reaction was incomplete (See Supporting Information).

**Figure 4:**
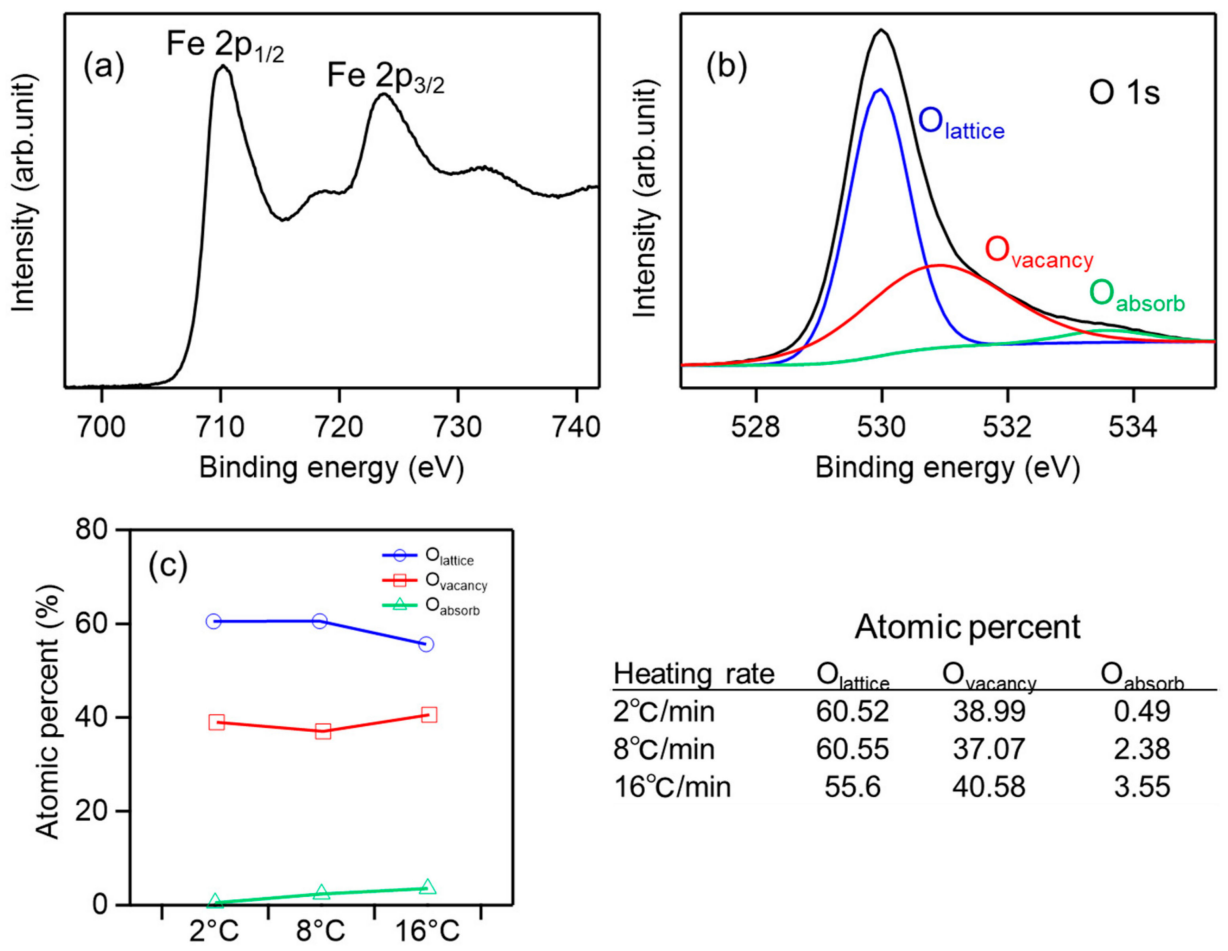
Representative XPS analysis (heating rate: 16 °C/min). (**a**) Fe 2p peak, (**b**) O 1s peak. The experimental O1s peak (black line) was divided into three components, and curve fitted. (**c**) The atomic percent calculated by the relative area ratio of the fitted curves as shown in (**b**). The table indicated the analysis of relative area ratio for all samples.

Figure 4 depicts representative XPS analysis results. Figure 4a illustrates the Fe 2p spectrum. The subtle variations in these peaks between samples render them challenging to evaluate. Consequently, this study focuses on analyzing the O 1s peak in Figure 4b. The 531 eV peak (red line) has been attributed to oxygen defects, and many studies have explored oxygen defects based on the peak area ratio [6,7,8,9,10,11,12,13,14,15,16]. Figure 4c summarizes how the area ratio of the three fitting curves, such as in Figure 4b, changes with the heating rate. Focusing on O_vacancy_, it is evident that the area ratio is maximized when the heating rate is set at 16 °C/min (40.58%), suggesting the generation of many defects on the surface.

**Figure 5:**
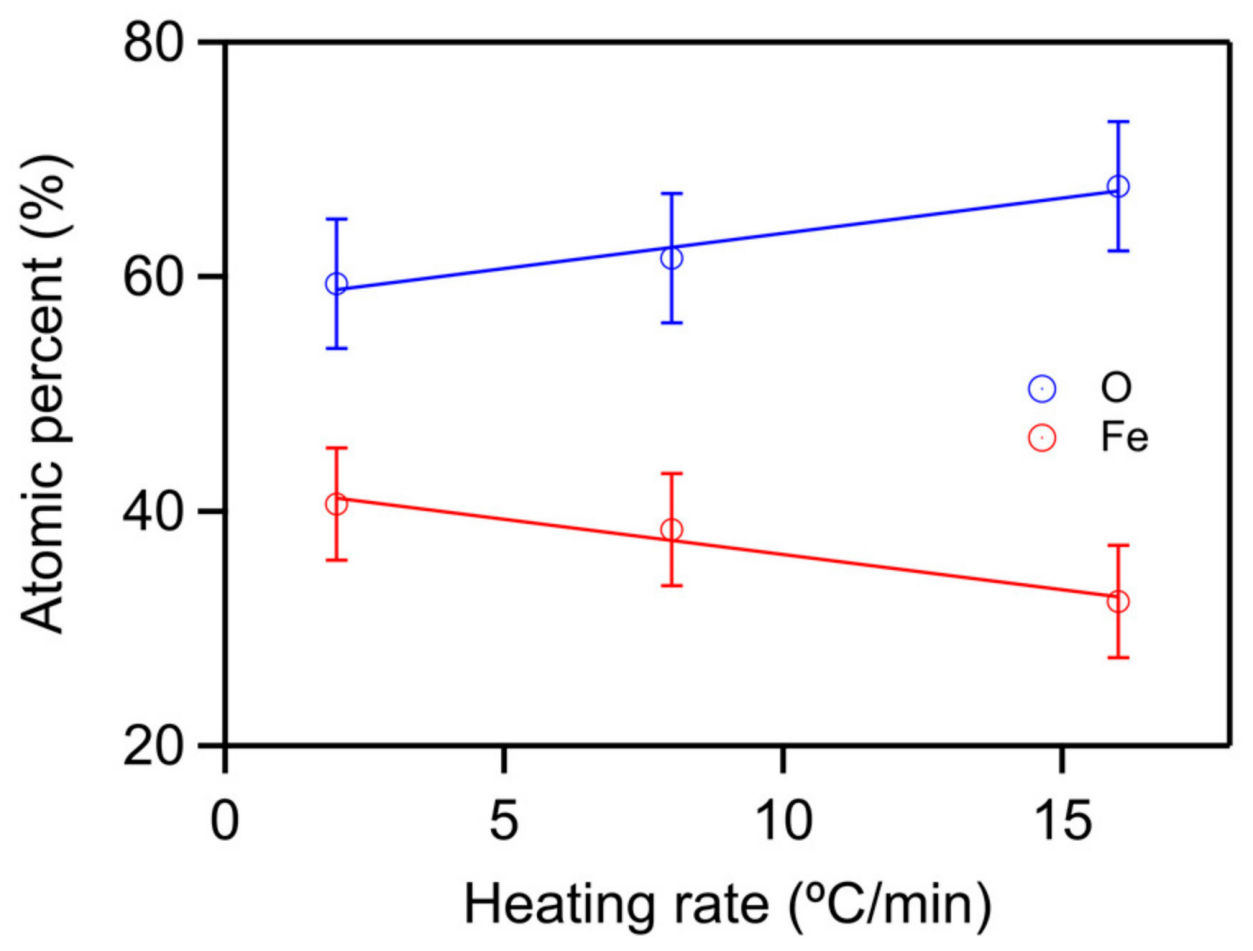
EDS analysis using an acceleration voltage of 8 keV (5 points average).

Figure 5 shows the results of the EDS analysis. These were measured using an acceleration voltage of 8 keV, so we believe that they also capture information from the interior of the particles. If we consider that the smaller the atomic ratio of O, the more oxygen defects are generated then the opposite of the XPS results is observed, and many defects are generated when the heating rate is the lowest at 2 °C/min. However, for photocatalysis, which is a surface reaction, surface defects will be more important than internal defects. The EDS information may be insightful for the application of other photodevices.

**Figure 6:**
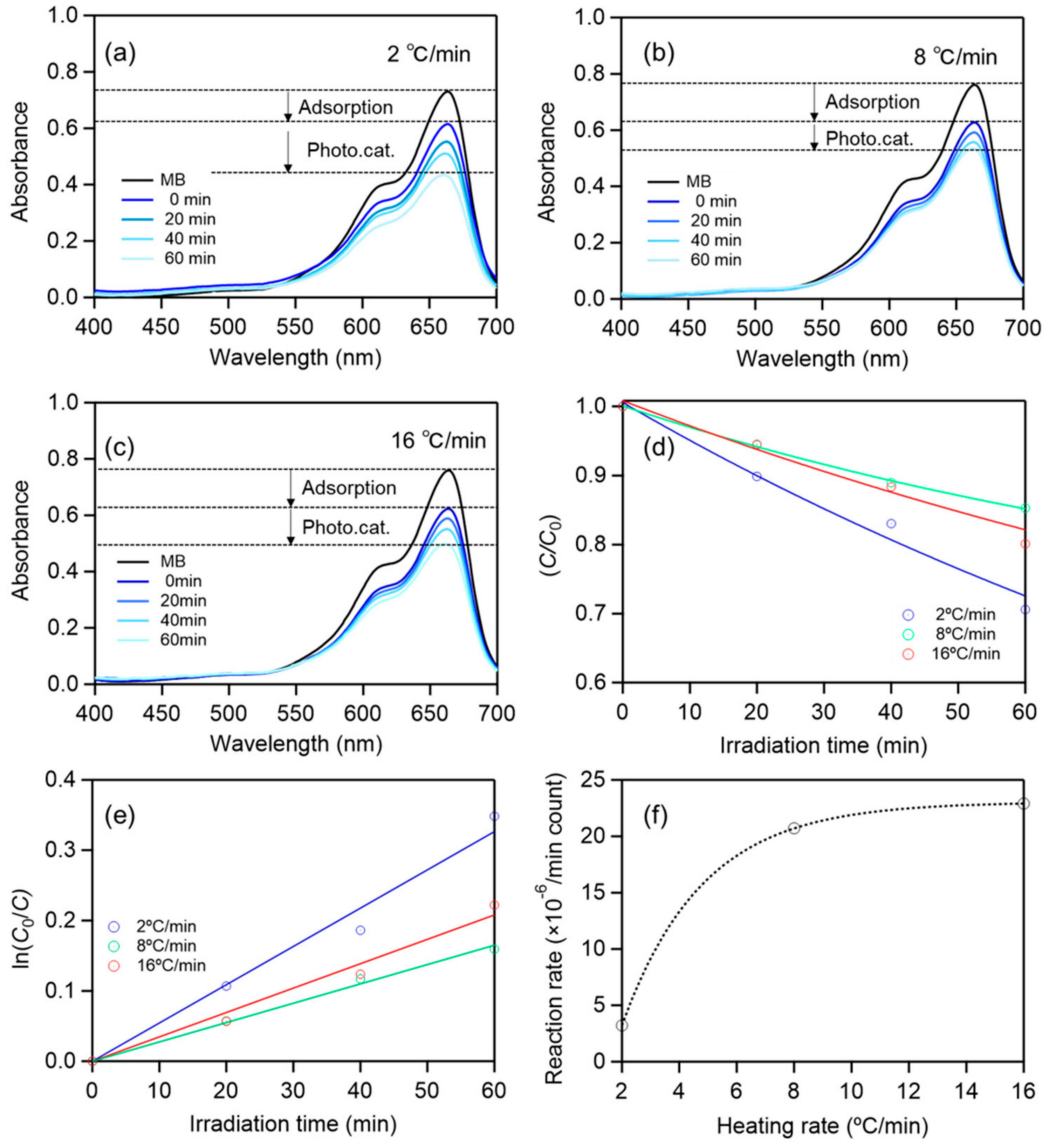
Photocatalytic performance. Absorbance intensity of MB: heating rate of (**a**) 2 °C/min, (**b**) 8 °C/min, (**c**) 16 °C/min, respectively. (**d**) Degradation rate of MB by measured absorbance spectra at 664 nm. (**e**) The plots of ln(*C*_0_/*C*) versus irradiation time. (**f**) Reaction rate per particle considering used number of hematite NPs.

Next, photocatalytic performance was evaluated by the decomposition of MB dye. Figure 6a–c shows the absorption spectra of MB. Before light irradiation, the suspended solution was stirred in the dark for 20 min. This stirring resulted in a decrease in the absorbance intensity due to the physical adsorption of MB molecules onto the surface of the NPs. Physical adsorption is also an important process for photocatalysis. Calculating the adsorption performance by the change in absorbance intensity, the adsorption rate was 15.8%, 17.6%, and 18.0%, respectively. Vo can also function as the site for the adsorption of dye molecules. Therefore, the difference in adsorption performance is reasonable and consistent with XPS results. Figure 6d,e show the change in absorbance peak intensity at 664 nm and linear fitting according to Equations (1) and (2). For a more accurate evaluation, the number of used hematite particles was calculated by considering the average particle size from the histogram, volume, and density. The reaction rate constant in Figure 6d,e is divided by the number of particles to calculate the reaction rate constant per particle. The results are shown in Figure 6f. These results show that the decomposition reaction rate increases as the heating rate increases, with a maximum difference of 7.1 times (from 3.22 × 10^−16^/min count to 2.29 × 10^−15^/min count, see Supporting Information).

## 4. Discussion

Here, we will discuss the following four factors: First, “what the heating rate affected and what it did not affect”. There were the effects of heating rate on the crystal size, shape, Vo distribution, and amount, as well as the dye adsorption and photocatalytic decomposition performance. It is generally known that the degree of crystal growth is sensitive to temperature, so it is important to pay attention to the size change during synthesis. On the other hand, there was no significant difference in crystallinity or micro-strain. In other words, it was found that the number of defects was not enough to lose the crystallinity or generate internal strain.

Second, we discuss “the reason why the Vo quantity differed depending on the heating rate”. In this study, we believe that the influence of heating under air is significant. That is, there is a lot of oxygen in the air, and the samples were heated in the presence of oxygen. Even if Vo is generated, if there is oxygen and sufficient heat energy, Vo will be compensated by oxygen in the air. Therefore, the longer the heating time, the more likely it is that Vo will disappear. If the heating rate is slow, the total heating time will simply be extended. Specifically, from room temperature (20 °C), it takes ca. 215 min, 54 min, and 27 min to reach 450 °C at heating rates of 2 °C/min, 8 °C/min, and 16 °C/min, respectively. Unsurprisingly, heating at 2 °C/min requires 8 times longer than at 16 °C/min, exposing the synthesized samples to high-temperature conditions. This factor was important in controlling the amount of Vo in the carbon reduction method.

Third, “the relationship between defects and photocatalytic performance”. To discuss the relationship between Vo and photocatalytic performance accurately, it is important to calculate the reaction rate constant per particle. In general, the absorption performance of light varies greatly depending on the size and shape based on plasmonics [24,25]. Therefore, we compared the amount of Vo measured by XPS and the photocatalytic performance of samples with similar sizes of 8 °C/min and 16 °C/min. The reaction rate constants per particle were 2.07 × 10^−5^ (8 °C/min) and 2.28 × 10^−5^ (16 °C/min), respectively. This is equivalent to an approximately 10% improvement in catalytic performance. According to XPS results of Figure 4c, the defect concentration was 37.07% and 40.58%, respectively. This ratio is also an approximately 10% increase in defect concentration. These results are well matched. Therefore, it is considered that the increase in the surface defect concentration improved the efficiency of photocatalytic performance due to an increase in adsorption performance and an increase in n-type electron carriers generation. 

Fourth, “prospects and challenges”. In this study, we attempted to manipulate the distribution of Vo content via controlled heating rates. However, limitations in the apparatus restricted our experiments to heating rates below 16 °C/min. Further investigation is necessary to determine if optimal heating rates exist for maximizing photocatalytic performance. Furthermore, persistent defect generation can induce a phase transformation to magnetite, necessitating the definition of clear boundary conditions for defect engineering within this system. Additionally, the inherent instability of the introduced Vo, susceptible to scavenging by environmental O_2_ and H_2_O, dictates further inquiries into defect persistence and retention strategies. However, the simplicity of hydrothermal synthesis and heating rate control as defective engineering tools remains noteworthy. Future exploration of this methodology holds significant promise for enhancing the performance of diverse semiconductor photocatalysts.

## 5. Conclusions

In summary, we investigated the introduction of Vo defects using hydrothermal synthesis. Particularly, we focused on carbon reduction reactions with different heating rates. As a result, the heating rate changed the crystal grain growth (size), shape, Vo distribution, and amount, while the crystallinity and micro-strain did not change. The surface Vo amount detected by XPS was the largest at the highest heating rate (16 °C/min). The physical adsorption performance of dyes and photocatalytic decomposition performance was also the largest, with a decomposition performance 7.1 times higher than that at the slowest heating rate (2 °C/min). The main reason for this is that overheating is performed in the air, so oxygen defects can always be compensated by oxygen in the air. As for Vo, it has been reported that Vo works as a carrier recombination site, while an increase in adsorption performance and an increase in n-type electron carriers due to defect generation. Therefore, it is crucial to investigate the boundary between the two factors. Thus, controlling the heating rate emerges as a key factor for optimizing future hematite photocatalyst synthesis and achieving enhanced activity.

## Data Availability

Data are contained within the article.

## Supplementary Materials

The following supporting information can be downloaded at https://www.mdpi.com/article/10.3390/ma17020395/s1. Figure S1. XRD patterns of hematite NPs synthesized at 350 °C via carbon-thermic reduction. Table S1. the information for calculation of reaction rate per minute and particle.
