# Supplementary material for: Hematite (α-Fe2O3) with Oxygen Defects: The Effect of Heating Rate for Photocatalytic Performance
Source: Materials (Basel). 2024 Jan 12;17(2):395. doi: 10.3390/ma17020395 (PMC10820844; doi:10.3390/ma17020395)
Supplement: Supplementary file 1 [file materials-17-00395-s001.zip › materials-2775128-supplementary.pdf]

Supporting Information

Influence of temperature for carbon-thermic process

Hematite was synthesized with the presence of PAA at 350 °C. However, the resulting product was a mixture of Fe_3O_4 and hematite, suggesting that the energy provided at 350 °C was insufficient for complete hematite formation. In contrast, the energy input of 450 °C was adequate for hematite synthesis, as evident from Figure 2a in the main manuscript.

(The synthesis experiment at 16°C/min was omitted from Figure S1 because it was deemed that the heat energy of 350°C was insufficient for the synthesis.)

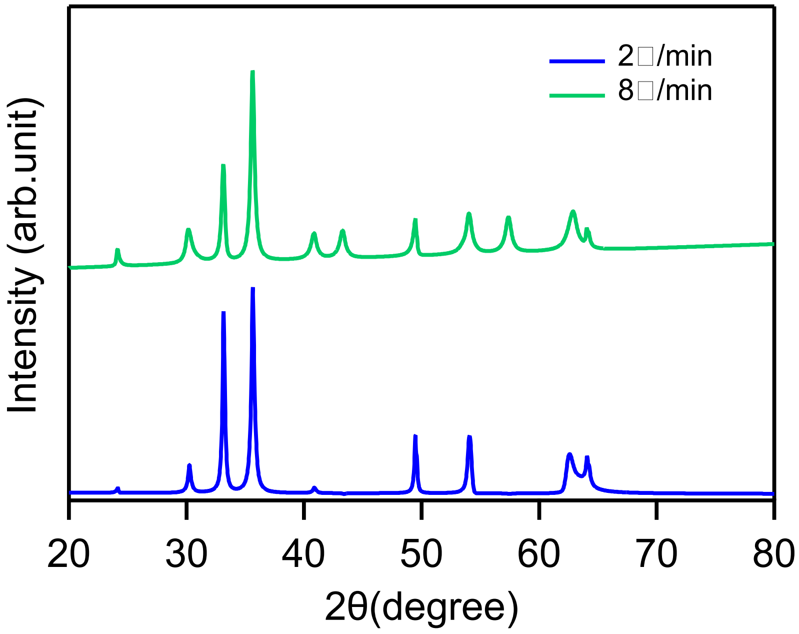

Figure S1. XRD patterns of hematite NPs synthesized at 350°C via carbon-thermic reduction.

The calculation of reaction rate per minute and particle

The particle sizes of the synthesized samples were different (Figure 1 in main manuscript). Therefore, when using an equal mass (10 mg) of hematite particles in experiments, the actual used number of hematite particles can vary significantly. To accurately calculate the photocatalytic reaction rate constant, it is necessary to determine the number of hematite particles used and compare the reaction rate constant per particle. The following table summarizes the information used in the calculations.

Table. S1 the information for calculation of reaction rate per minute and particle.

heating rate	rection rate [/min]	density (mg/cm^3)	size (cm)	Volume(cm^3/count)
2°C/min	0.00544	5.24E+03	3.00E-06	1.13E-16
8°C/min	0.00275	5.24E+03	7.00E-06	1.44E-15
16°C/min	0.00347	5.24E+03	6.70E-06	1.26E-15

heating rate	mass of used photo.cat.(mg)	number of particles	reaction rate (/min count)
2°C/min	10	1.69E+13	3.22E-16
8°C/min	10	1.33E+12	2.07E-15
16°C/min	10	1.51E+12	2.29E-15
